# Investigation on three-dimensional printed prosthetics leg sockets coated with different reinforcement materials: analysis on mechanical strength and microstructural

**DOI:** 10.1038/s41598-024-57454-8

**Published:** 2024-03-21

**Authors:** Muhammad Hanif Ramlee, Muhammad Imam Ammarullah, Nurelisya Suraya Mohd Sukri, Nur Syafiqah Faidzul Hassan, Muhammad Hanif Baharuddin, Mohammed Rafiq Abdul Kadir

**Affiliations:** 1 Bone Biomechanics Laboratory (BBL), Department of Biomedical Engineering and Health Sciences, Faculty of Electrical Engineering, Universiti Teknologi Malaysia, Johor Bahru 81310, Johor, Malaysia; 2 Bioinspired Devices and Tissue Engineering (BIOINSPIRA) Research Group, Universiti Teknologi Malaysia, Johor Bahru 81310, Johor, Malaysia; 3 Department of Mechanical Engineering, Faculty of Engineering, Universitas Diponegoro, Semarang, 50275 Central Java Indonesia; 4 Undip Biomechanics Engineering and Research Centre (UBM-ERC), Universitas Diponegoro, Semarang, 50275 Central Java Indonesia; 5 Department of Mechanical Engineering, Faculty of Engineering, Universitas Pasundan, Bandung, 40153 West Java Indonesia; 6 Biomechanics and Biomedics Engineering Research Centre, Universitas Pasundan, Bandung, 40153 West Java Indonesia; 7 Department of Biomedical Engineering, Faculty of Engineering, Universiti Malaya, 50603 Kuala Lumpur, Federal Territory of Kuala Lumpur Malaysia

**Keywords:** 3D printing, Prosthetics leg, Post-processing, Reinforcement materials, Artificial leg, Health care, Materials science

## Abstract

Previous research has primarily focused on pre-processing parameters such as design, material selection, and printing techniques to improve the strength of 3D-printed prosthetic leg sockets. However, these methods fail to address the major challenges that arise post-printing, namely failures at the distal end of the socket and susceptibility to shear failure. Addressing this gap, the study aims to enhance the mechanical properties of 3D-printed prosthetic leg sockets through post-processing techniques. Fifteen PLA + prosthetic leg sockets are fabricated and reinforced with four materials: carbon fiber, carbon-Kevlar fiber, fiberglass, and cement. Mechanical and microstructural properties of the sockets are evaluated through axial compression testing and scanning electron microscopy (SEM). Results highlight superior attributes of cement-reinforced sockets, exhibiting significantly higher yield strength (up to 89.57% more than counterparts) and higher Young’s modulus (up to 76.15% greater). SEM reveals correlations between microstructural properties and socket strength. These findings deepen the comprehension of 3D-printed prosthetic leg socket post-processing, presenting optimization prospects. Future research can focus on refining fabrication techniques, exploring alternative reinforcement materials, and investigating the long-term durability and functionality of post-processed 3D-printed prosthetic leg sockets.

## Introduction

A prosthetic leg socket is a structural link between an amputee’s residual limb and the prosthesis. Currently, where the complexity of the conventional method to manufacture prosthetic leg sockets is still a significant hurdle, there are promising prospects for integrating additive manufacturing (AM) as a fabrication process^1^. AM is a digital manufacturing technology that enables the creation of three-dimensional (3D) printed models or functional objects with sophisticated geometries^2–5^. To fabricate a 3D-printed prosthetic leg socket, a 3D scanner generates a positive mold of the residual limb, creating a 3D model that can be altered in computer-aided design (CAD) software and printed with a 3D printer^6^. Compared to the conventional approach, AM is the preferred method for creating customized leg sockets due to its ability for rapid production, resulting in reduced fabrication time and lower labor and material costs^7^. AM’s ability to decrease the time required to create a perfect socket fit is especially advantageous for patients with unstable limb volumes who require multiple socket modifications^8^. In addition, the cost of a 3D printer and filament materials for 3D printing is significantly lower than that of conventional manufacturing techniques, making them a viable option for use in developing countries^9^.

To meet users’ needs regarding function and ergonomic, developing strong, durable, and dependable prosthetic leg sockets is essential to enable users to maintain an independent lifestyle and actively participate in daily tasks^10^. Despite the benefits of adopting 3D printing technology in socket manufacturing, it is still being determined to what extent 3D printing can yield sockets with sufficient ultimate forces for safe, long-term use^11^. Although several studies investigate the effectiveness of 3D-printed prosthetic leg sockets in clinical practice, they are frequently conducted in a short period, implying that their findings need to be more conclusive^9^. Regardless of significant technological advancements, such as the development of more robust materials or composites with high toughness, stiffness, and improved printing parameters, there is a lack of research on the ultimate force and durability of 3D-printed leg sockets, which may make prosthetists less confident in their use^11^.

On top of that, there is currently no test standard for evaluating the mechanical properties of 3D-printed prosthetic sockets. However, prosthetic leg socket strength assessment is commonly carried out following the International Organization for Standardization (ISO) 10,328: Prosthetics–Structural testing of lower-limb prostheses^12^. This standard guarantees the reliability of prosthetic leg sockets for patient usage by conducting mechanical testing under maximum loading conditions, typically during the heel-strike or toe-off positions^13^. Consequently, failures in 3D-printed prosthetic leg sockets are predominantly observed at the distal end of the socket or the pyramid attachment, aligning with the specifications of the ISO testing standard^11^. Addressing this, researchers proposed reinforcing the distal socket with a composite infill approach to enhance socket strength^14^. An additional concern regarding 3D-printed prosthetic leg sockets stems from their susceptibility to shear failure due to the inherent layer-by-layer structure^12^. However, it is possible to reduce shear failure by adjusting various printing parameters, such as raster angle, infill density, infill pattern, and the inclusion of corrugations^15^. Nevertheless, the fundamental filament deposition method remains unaltered, indicating that the ongoing risk persists.

Another prominent feature impacting the strength of a prosthetic leg socket is the choice of socket materials and fabrication. Over the years, polylactic acid (PLA), polypropylene (PP), and polycaprolactone (PCL) have been touted as reliable sources for fused filament fabrication (FFF) print materials^16^. In efforts to bolster the strength and toughness of 3D-printed prosthetic leg sockets, researchers have turned to the use of carbon-reinforced and glass-reinforced composite polymer filaments^6,14^. Furthermore, utilizing carbon fiber composite during 3D printing has shown notable enhancements in layer-on-layer adhesion^17^. In conventional prosthetic leg socket fabrication, various material layups were discovered to improve the strength of laminated composite sockets (LCS)^11^. Similarly, the wet layup approach, leveraging interwoven carbon, Kevlar, or glass fibers, has demonstrated its effectiveness in enhancing the mechanical strength of LCS within the context of traditional socket fabrication^18^.

So far, research into the causes and solutions of prosthetic leg socket problems in additive manufacturing has primarily focused on aspects such as the fabrication method, design, printing parameters, and material selection—all of which are components of the pre-processing process. However, these methods still fail to address the significant challenges that arise post-printing: failures at the distal end of the socket and susceptibility to shear failure. Moreover, there has been little work to investigate the usefulness of the post-processing method in increasing the strength of the 3D-printed prosthetic leg socket. Hence, there is the possibility of emulating the strength-enhancing properties in post-processing steps using a wet layup approach to reinforce and improve the mechanical properties of 3D-printed prosthetics leg sockets^18^. One of the methods for the reinforcement is surface coatings with various materials that could achieve specific visual and mechanical properties^19^. According to Poornaganti et al.^20^ from their previous studies on the surface characterization on 3D printed model, they found that the additional reinforcement materials on the surface may improve properties such as dimensional accuracy, water absorption, surface texture and wettability. Another study by Vicente et al.^21^ found that protective coatings such as polyurethane (PU) wood sealant and acrylic aqueous varnish were the most effective materials in preventing water absorption to the 3D-printed parts thus may increase the mechanical properties up to 24% in respect to the specimen strength and ductility/toughness.

Even though, some researchers have conducted many investigations on the surface coatings on the 3D-printed part, nevertheless, none of the previous studies very focus on the coating materials for the 3D-printed prosthetics leg socket, thus this made limitations on the understanding of mechanical properties and microstructural integrity of different reinforcement materials. Therefore, this study aimed to fabricate 3D-printed prosthetic leg sockets and reinforce them with reinforcement layer materials: carbon fiber, carbon-Kevlar fiber, fiberglass, and cement in which the mechanical properties and microstructural integrity were investigated further. The reinforced sockets were then subjected to axial mechanical testing to determine their mechanical performance, followed by scanning electron microscopy to characterize their microstructural properties. From the literature, the principal conclusion regarding the strongest material suggests that carbon fiber demonstrates superior strength attributes, as highlighted in various studies^22–25^. The ultimate strength of a 3D-printed prosthetic leg socket must be high enough to sustain the daily accumulation of loads and stresses to avoid mechanical failure. Ergo, developing and implementing suitable pre- and post-processing techniques is essential to enhance the overall quality, durability, and strength of 3D-printed prosthetic leg sockets.

## Materials and methods

### 3D printing for prosthetic leg socket

The design of the 3D model for the transtibial prosthetic socket was sourced from a prior study related to passive transtibial prosthetic legs^26^. Table 1 presents the specified design parameters during the development of the transtibial prosthetic socket. Next, the transtibial prosthetic leg socket design was exported as an STL file to Ultimaker Cura (Ultimaker, Utrecht, Netherlands) for slicing and optimizing printing parameters to ensure the best possible outcome during the 3D printing process.

**Table 1:** Transtibial prosthetic leg socket design parameters.

Parameters	Value
Height	173 mm
Diameter	90 mm
Thickness	6 mm
Material	Polylactic acid (PLA +)

**Figure 1:**
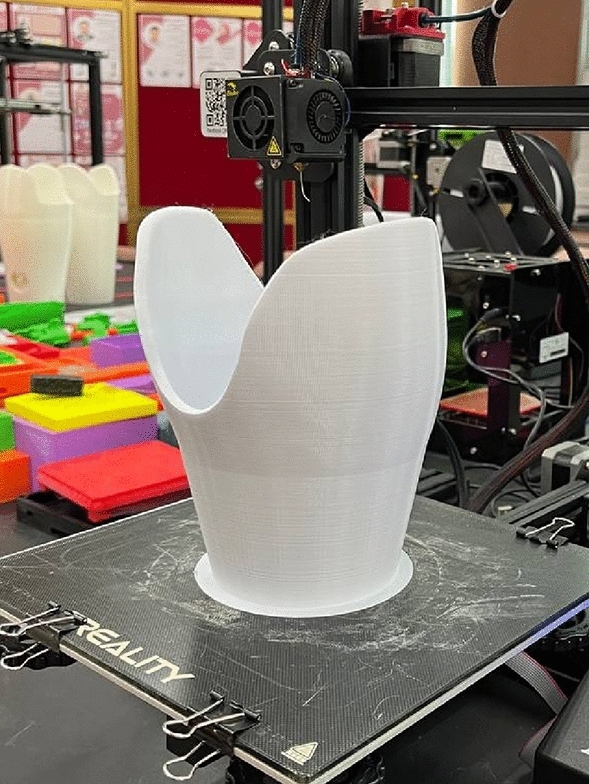
3D-printed PLA + transtibial prosthetic leg socket.

Fifteen prosthetic leg sockets were 3D-printed using eSUN polylactic acid plus (PLA +) filament (eSUN3D, Shenzhen, China), as depicted in Fig. 1. Table 2 shows the printing parameters utilized were derived from the filament manufacturer’s guidelines. The infill density of the prosthetic leg sockets is set to 40% to improve mechanical properties^27^. Additionally, the infill pattern chosen for the sockets is tri-hexagon, as it offers advantages in strength and mechanical properties^28^.

**Table 2:** Transtibial prosthetic leg socket design parameters.

Parameters	Value
Extruder temperature	210 °C
Bed temperature	60 °C
Infill pattern	Tri-hexagon
Infill density	40%
Layer height	0.2 mm
Layer width	0.4 mm
Top thickness	1.2 mm
Bottom thickness	1.2 mm
Top layer	6
Bottom layer	6
Wall thickness	4.0 mm
Print speed	55 mm/s or slower
Build plate adhesion	Brim

### Post-processing process for prosthetic leg socket

Three post-processing steps were executed on all fifteen prosthetic leg sockets to enhance the aesthetic appearance and proper adhesion to the reinforcement layer materials^29,30^. These steps comprised support removal, surface sanding for smoothing, and the application of epoxy resin as a surface treatment. Table 3 below displays the specified parameters to ensure consistency for carbon, carbon-Kevlar, and fiberglass fiber reinforcements. The fiber-to-matrix ratio was established by weighing each epoxy resin layer against the three distinct fibers^31^.

**Table 3:** Fiber reinforcement parameters.

Fiber parameters	Value
Thickness	0.3 mm
Pattern	Twill weave
Fiber-to-matrix (FTM) ratio	~ 30:70

Meanwhile, Table 4 presents the specified parameter for cement-sprayed sockets. While it would have been preferable for the cement socket’s thickness to align with that of the fabric, it was observed that beyond the fourth layer, the cement tended to peel off at the edges, resulting in cracking and disintegration. As a result, the maximum number of sprayed coatings was limited to four, with the topcoat being applied twice to seal the underlying cement layers.

**Table 4:** Cement reinforcement parameters.

Cement parameters	Value
Thickness of the cement spray	0.10 mm (4 layers)
Thickness of the top coat	0.04 mm (2 layers)

#### Support removal

The support brim of the 3D-printed socket was manually removed by hand. In cases where the support did not come out in one piece or if any remnants were left, pliers or tweezers were used to remove the remaining pieces. The support of the 3D-printed part has been cut with the cutter within the boundary of the desired prosthetics socket.

#### Sanding

**Figure 2:**
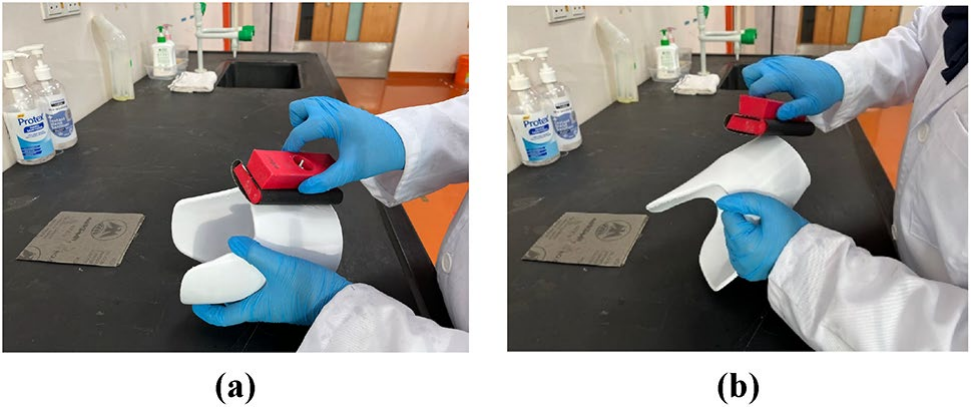
Sanding the 3D-printed sockets: (**a**) Socket surface; (**b**) Socket edge.

The 3D-printed prosthetic leg sockets exhibited slightly rough surfaces near the edges and where the prints meet along the back socket circumference. To achieve a smoother finish, sanding process performed to even out the layer lines and rough edges to achieve a smoother finish, as shown in Fig. 2. The process was executed using coarser grain size sandpapers (P400) first before gradually progressing to finer sizes (P600, P800, P1000), using circular motions to ensure an even smoothing of the surface and edges of the socket as suggested by previous study^32^. Precautions were taken into consideration during this process, where this is important to avoid prolonged sanding in a single area, as this could generate heat or potentially alter the dimensions of the prototype. After sanding, the parts were carefully wiped down with a dry cloth to remove any remaining fine dust particles. They were then wiped down again with a thin layer of acetone to ensure a clean and smooth surface, effectively removing any lingering dust particles.

#### Surface treatment

**Figure 3:**
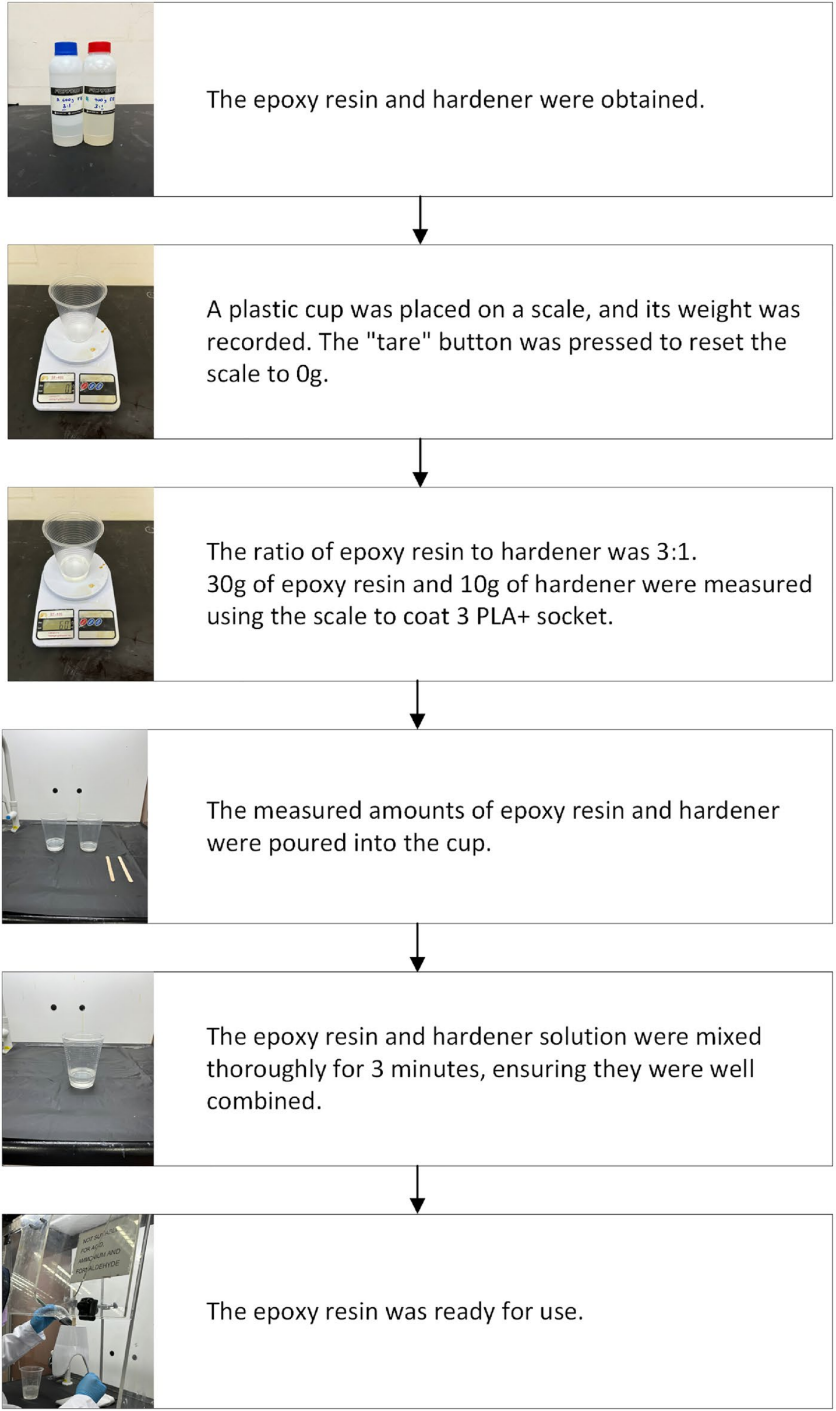
Steps to prepare two parts epoxy resin.

Finally, a single layer of epoxy resin was applied to the exterior surface of the socket to strengthen it and aid bonding with other materials^33,34^. This treatment protects the sockets from moisture, chemicals, and UV radiation^35^. First, the epoxy resin and hardener were weighted where the ratio was 3:1 or 75% volume fracture. Later, both of them were mixed together into a cup for 3 min by manually stirring. Then, the final step is to brush the mixed chemicals onto the surface of 3D-printed prosthetics socket. Figure 3 summarize the flow on how to prepare the two-part epoxy resin for surface treatment.

### Reinforcement layer for prosthetic leg socket

After post-processing, four groups of three sockets will be reinforced with carbon fiber, carbon-Kevlar, fiberglass, and cement. The fabric lamination process will follow the hand layup process outlined in a previous study about prosthetic foot lamination ^23^. Meanwhile, the cement coating will adhere to the manufacturer’s protocol, ensuring the cement layer is maximized to its total capacity. To create five different socket reinforcement groups, three sockets were reinforced with four different reinforcement materials and a control group.

#### Carbon fiber

**Figure 4:**
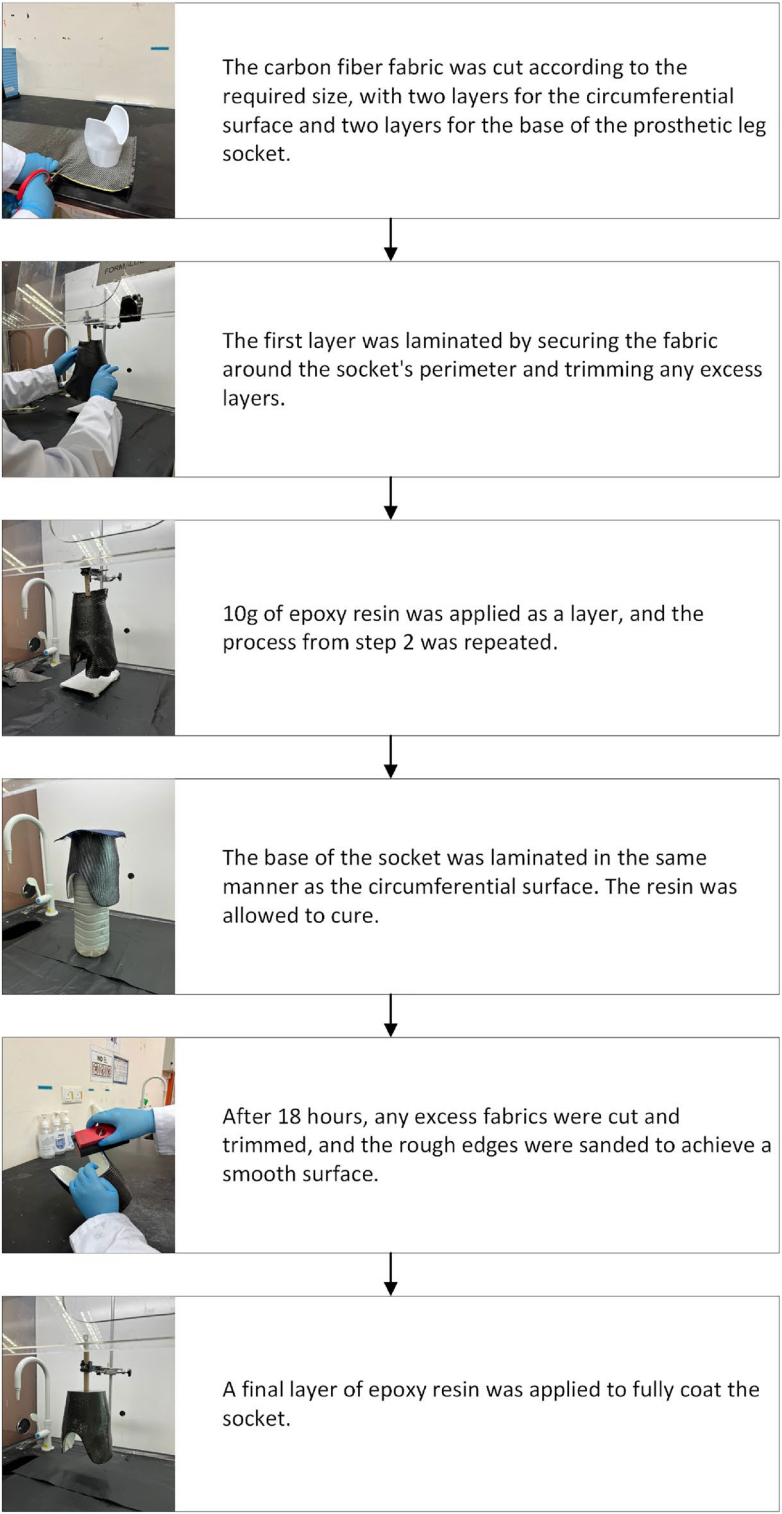
Step-by-step process detailing the lamination of carbon fiber fabric onto a leg socket.

The hand layup method was used to reinforce the carbon fiber fabric onto the prosthetic leg socket^23^. Figure 4 illustrates the step-by-step procedure for laminating a layer of carbon fiber fabric onto the leg socket.

A second layer of carbon fiber fabric was applied by following the same steps as above. Subsequently, the sockets were allowed to dry before trimming, sanding, and receiving a final epoxy resin coating to enhance protection and durability.^33^.

#### Carbon-Kevlar fiber

The method described in Section "Carbon fiber" was applied to three prosthetic leg sockets for the carbon-Kevlar fiber reinforcement.

#### Fiberglass

Similarly, the same method outlined in Section "Carbon fiber" was utilized for the application of fiberglass fiber fabric onto the prosthetic leg sockets.

#### Cement

**Figure 5:**
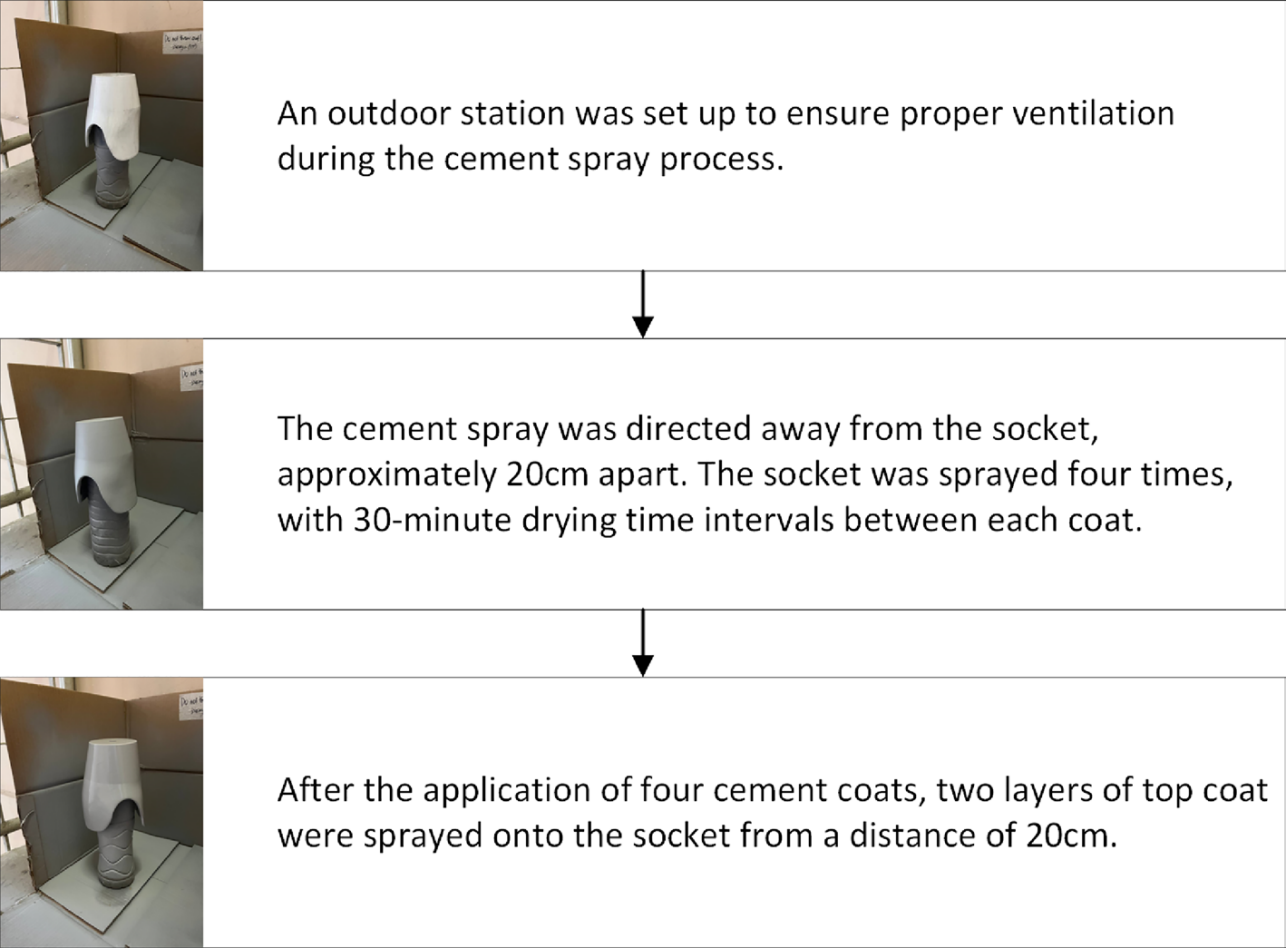
Step-by-step process illustrating the reinforcement of a cement layer onto a leg socket.

Figure 5 visually depicts the newly established step-by-step process for applying cement onto a prosthetic leg socket through spraying. This protocol is new and not sourced from any previous study.

### Prototype evaluation

#### Axial compression testing

**Figure 6:**
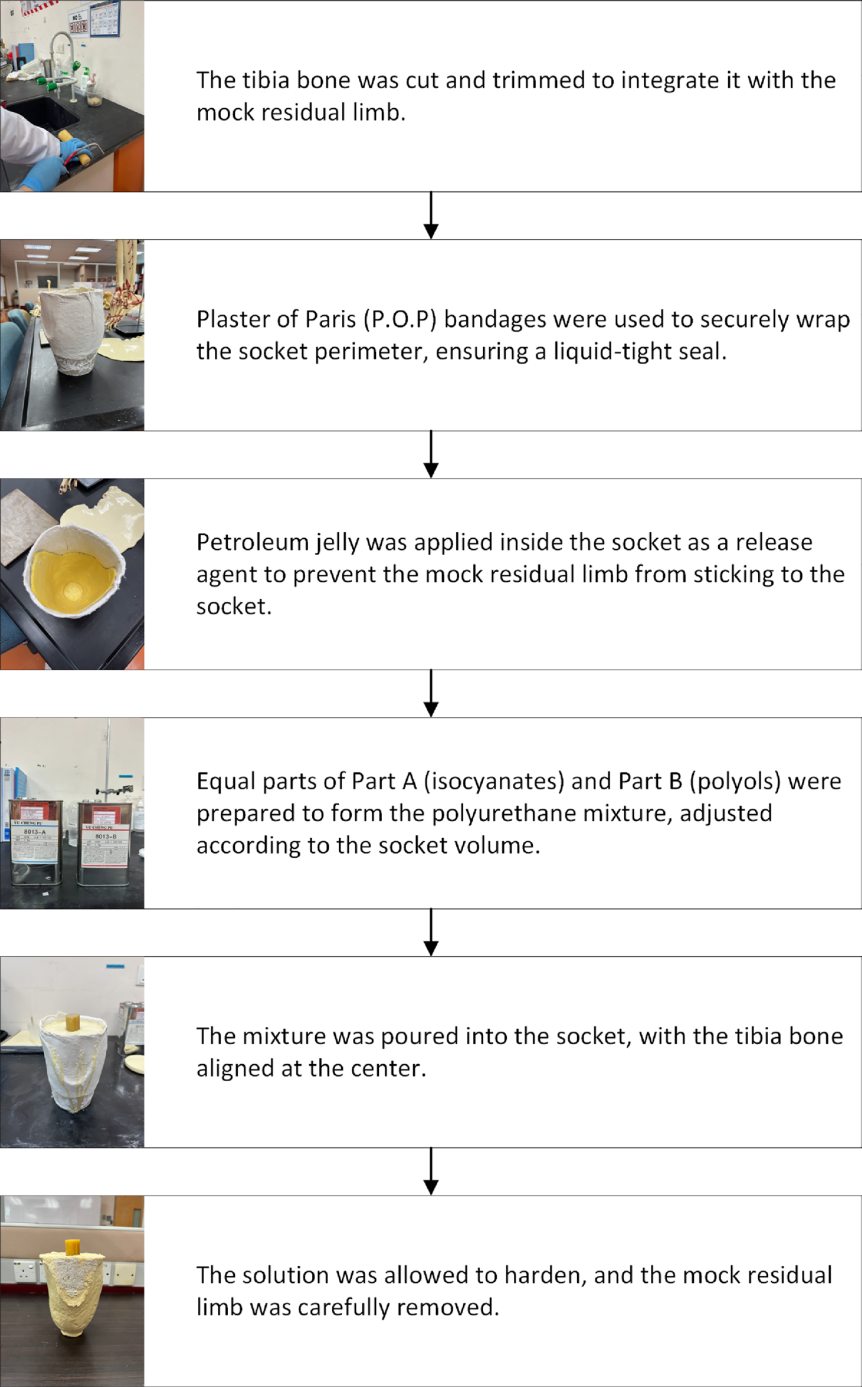
Step-by-step process illustrating the reinforcement of a cement layer onto a leg socket.

For mechanical testing, a mock residual limb was fabricated to replicate the secure attachment of an amputated leg to the socket. Figure 6 presents the step-by-step procedures for creating the mock residual limb^26^.

**Figure 7:**
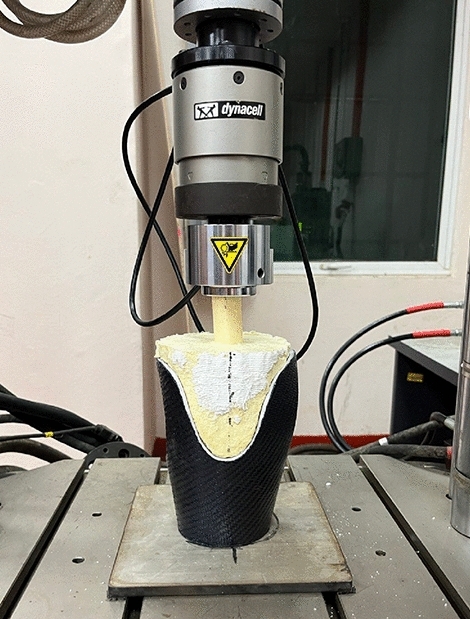
Test setup used for axial compression testing.

The socket samples underwent axial compression testing using an Instron® 8874 Series universal testing machine (Illinois Tool Works Inc., Norwood, United States), following the guidelines established for axial compression of FRP-confined concrete-core-encased rebar^36^. The axial compression test involved applying a downward force to the socket at a 3 mm/min rate, simulating the load applied on the prosthetic device during the mid-stance position^26^. As the testing progressed, the loading force reached a plateau, indicating that the machine could no longer compress the socket further. Testing concluded one minute after a constant reading was obtained for further analysis. Figure 7 depicts the setup utilized for the axial compression testing.

#### Scanning electron microscopy (SEM)

**Figure 8:**
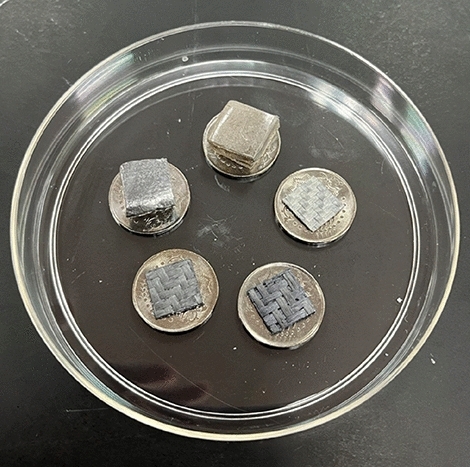
Gold-sputtered reinforcement layer samples.

SEM analysis was conducted on samples from each reinforced leg socket with different reinforcement materials. Firstly, the samples were sectioned into dimensions of 1 × 1 × 1cm and coated with a thin layer of gold for 20 min to enhance conductivity, as depicted in Fig. 8^37^. Subsequently, SEM imaging was performed with a voltage of 15 kV using a Hitachi TM3000 Tabletop Scanning Electron Microscope machine (Hitachi, Ltd., Ibaraki, Japan) at various magnifications: 50x, 250x, 500x, and 1.5kx for fiber specimens, and 1.5kx, 2.5kx, and 5kx for cement and control specimens. The obtained images were then processed using the built-in software to enhance resolution and clarity.

## Results

### Reinforced 3D-printed prosthetic leg socket

#### Carbon fiber

**Figure 9:**
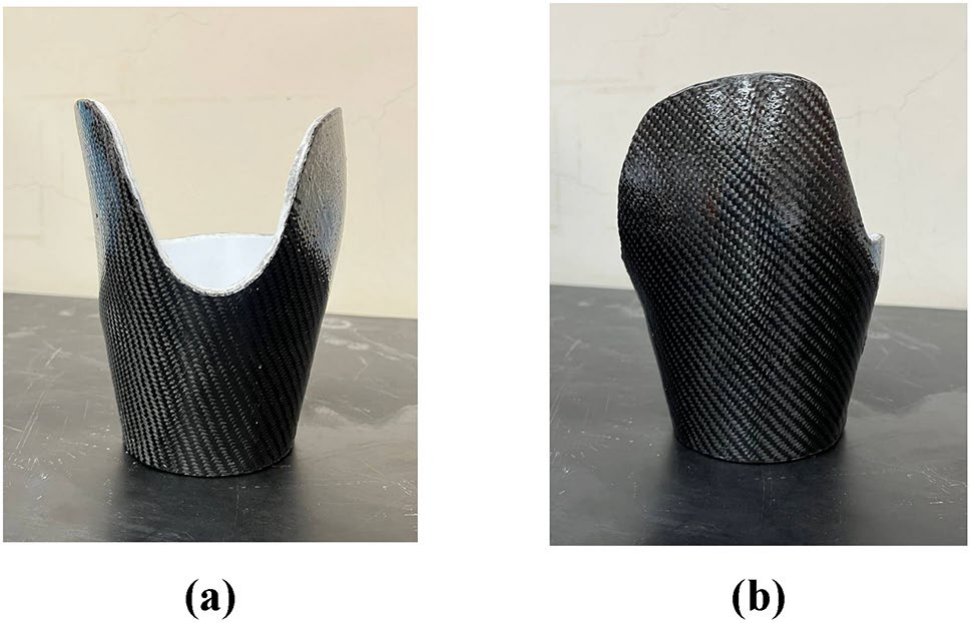
Carbon fiber reinforced 3D-printed prosthetic leg socket: (**a**) Front view; (**b**) Side view.

Figure 9 shows the carbon fiber-reinforced 3D-printed prosthetic leg socket.

#### Carbon-Kevlar fiber

**Figure 10:**
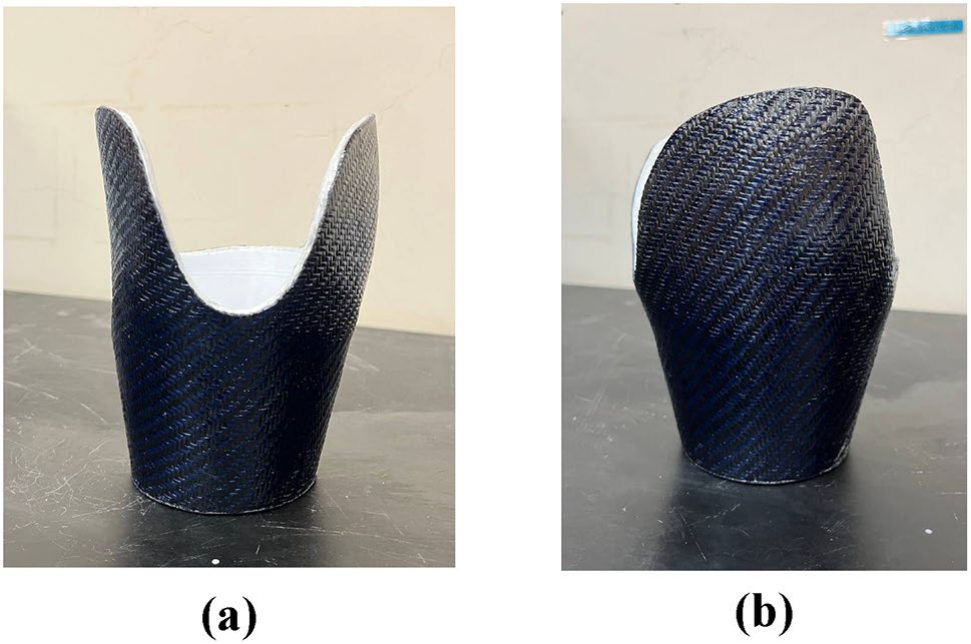
Carbon-Kevlar fiber reinforced 3D-printed prosthetic leg socket: (**a**) Front view; (**b**) Side view.

Figure 10 shows the carbon-Kevlar fiber-reinforced 3D-printed prosthetic leg socket.

#### Fiberglass

**Figure 11:**
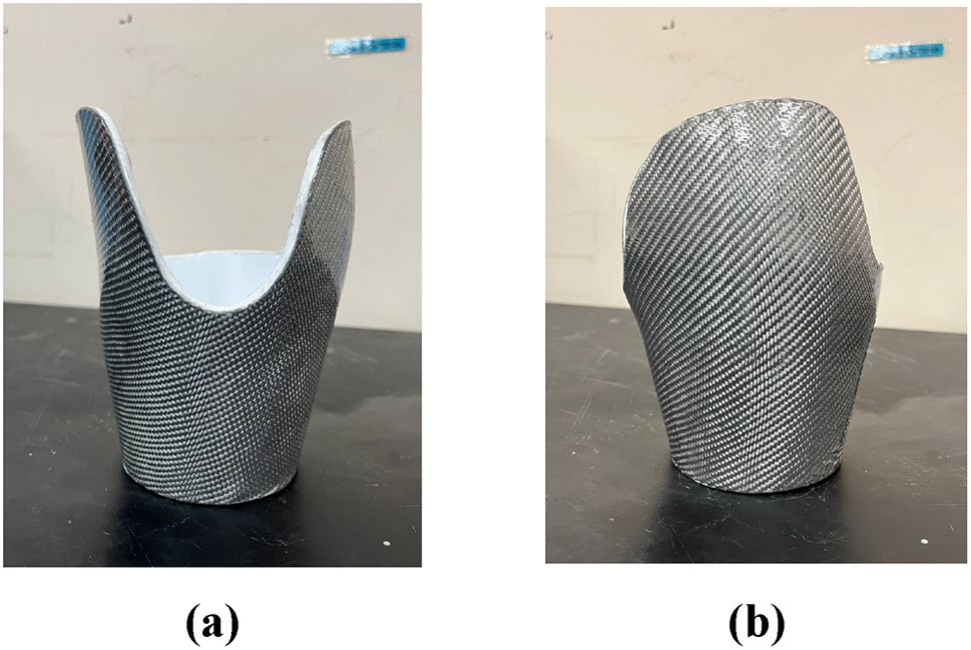
Fiberglass reinforced 3D-printed prosthetic leg socket: (**a**) Front view; (**b**) Side view.

Figure 11 shows the fiberglass reinforced 3D-printed prosthetic leg socket.

#### Cement

**Figure 12:**
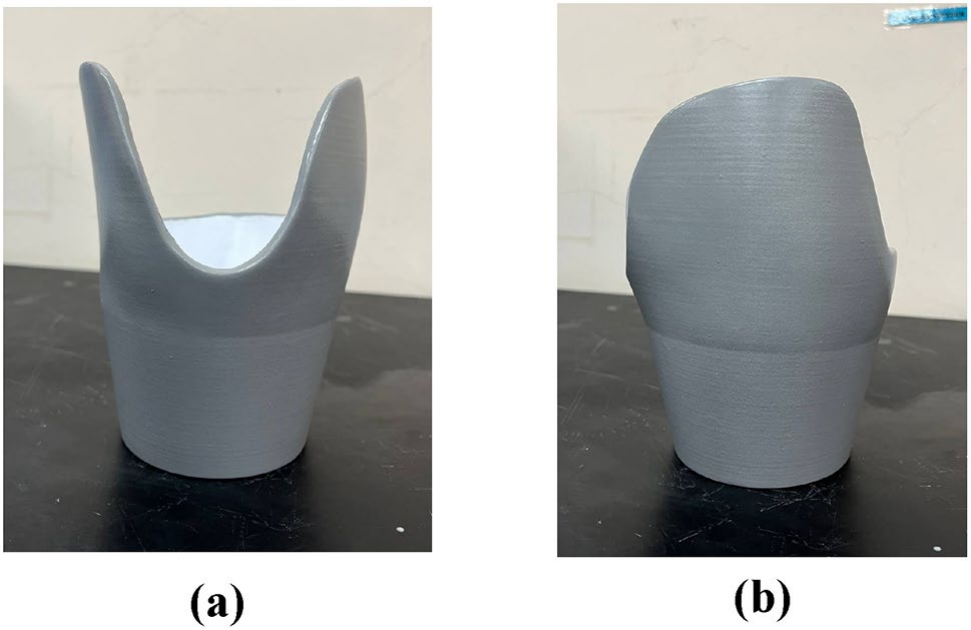
Cement reinforced 3D-printed prosthetic leg socket: (**a**) Front view**;** (**b**) Side view.

Figure 12 shows the cement-coated 3D-printed prosthetic leg socket.

#### Control

**Figure 13:**
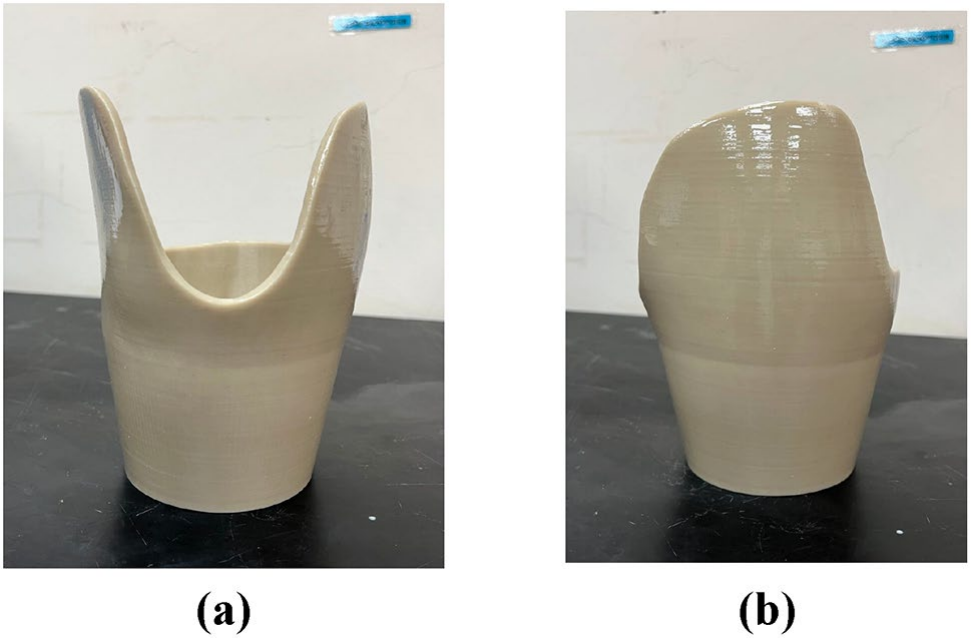
Control group of 3D-printed prosthetic leg socket: (**a**) Front view; (**b**) Side view.

Figure 13 shows the control 3D-printed prosthetic leg socket.

### Axial compression testing

#### Stress–strain curve

The values obtained during the axial compression testing included load (N), extension (mm), and time (s). To calculate stress, the load is divided by the socket’s base area of 6.3617 × 10–3 mm^2^, while the strain is obtained by dividing the extension by 173 mm. The stress–strain curves are plotted for all samples to determine each socket type’s yield strength and Young’s modulus. The analysis aimed to address the following research questions: (1) How does the strength of the reinforcement materials differ from each other? (2) What is the best reinforcement material in terms of strength? (3) How significant are the differences between each group?

**Figure 14:**
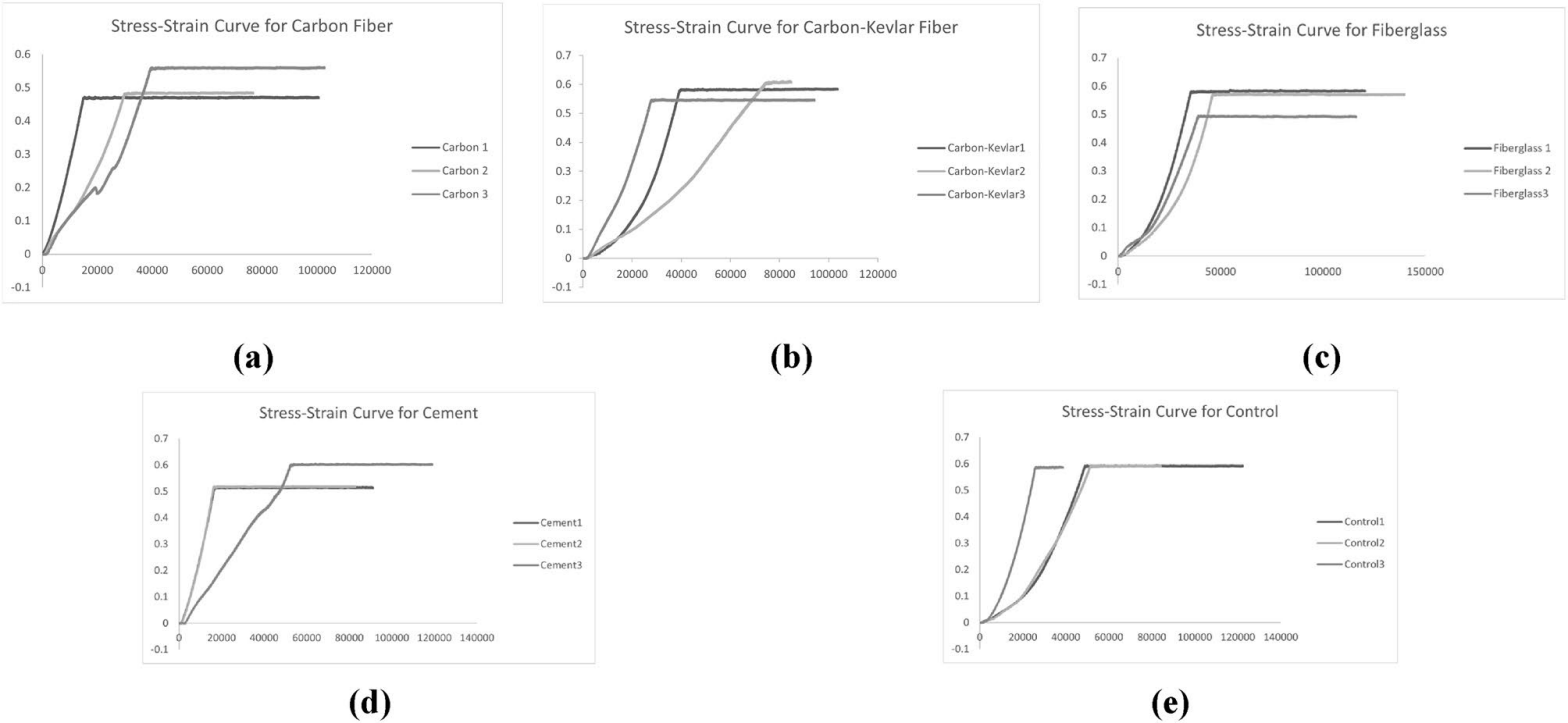
Stress–strain curve: (**a**) Carbon Fiber; (**b**) Carbon-Kevlar Fiber; (**c**) Fiberglass; (**d**) Cement; (**e**) Control.

Figure 14 illustrates the stress–strain curves for all groups of reinforced sockets, including the control. To determine the yield strength and Young’s modulus, identifying a linear initial slope through linear regression is necessary to obtain the slope coefficient^38^. However, this method is not feasible due to a slightly curved elastic region. Instead, the yield strength and Young’s modulus were ascertained by plotting the initial slope before the curve reached a plateau as an approximation^39^.

In Fig. 14a depicting carbon fiber reinforced sockets, Samples 1 and 2 exhibit similar behavior, while Sample 3 displays some deviation in the initial stress–strain region. Next, Fig. 14b showcases the stress–strain curve for three carbon-Kevlar fiber reinforced sockets samples. The stress–strain curves exhibit variations among the three samples, suggesting unequal variance. It is important to note that while the obtained strength is lower than the standard strength, the results of the axial compression tests may not necessarily represent the typical or average properties of carbon-Kevlar fiber under different conditions. Figure 14c illustrates the stress–strain curve for fiberglass reinforced sockets. Like the previous reinforcement materials, the obtained yield strength and Young’s modulus were below the standard values. However, the samples exhibited similar behavior approaching the plastic region, indicating an equivalent level of socket quality within the group.

For cement-reinforced sockets in Fig. 14d, the first two samples exhibit identical stress–strain curves. However, the third sample has a less steep slope, although it possesses higher stress than the first two sockets. Lastly, Fig. 14e shows the stress–strain curves for the control sockets without a reinforcement layer. The second and third control sockets exhibited similar behavior in both the elastic and plastic regions. However, the first socket had a steeper slope, indicating it was stiffer and more robust than the others.

#### Yield strength (σ_Y_)

**Figure 15:**
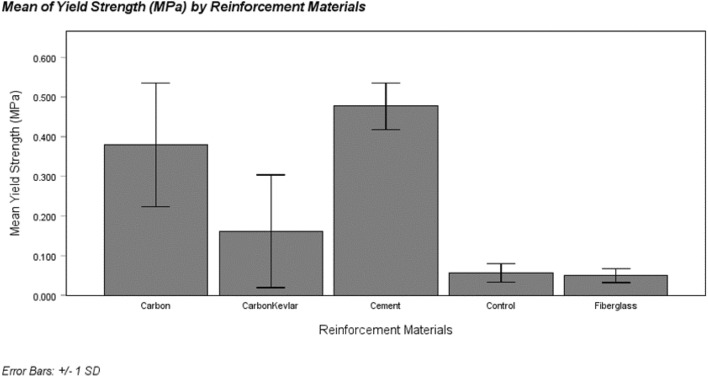
Mean of yield strength for different types of reinforced sockets.

Figure 15 presents the mean yield strength values for different socket reinforcement materials. The materials’ yield strength in descending order are cement, carbon, carbon-Kevlar, fiberglass, and control. However, it is worth noting that the error bars associated with the data points are significant, indicating potential issues with sample size, quality, and testing errors. The chosen standard deviation for the error bars is 1. These results emphasize the importance of further investigation and improvement in experimental procedures to ensure more reliable and accurate yield strength measurements in future studies. Referring to Table 5 for the one-way ANOVA, it becomes evident that there are significant differences in yield strength among the group means (p < 0.05).

**Table 5:** One-way ANOVA for yield strength.

	Sum of squares	df	Mean square	F	Sig
Between groups	0.451	4	0.113	11.572	0.001
Within groups	0.097	10	0.010		
Total	0.549	14			

Following this, the Tukey’s Honest Significant Difference (HSD) test, as demonstrated in Table 6, is applied after a one-way ANOVA. This step is utilized to pinpoint and categorize the specific socket groups that exhibit significant differences from one another, enabling precise and valid comparisons^40^.

**Table 6:** Tukey’s honest significant difference (HSD) for yield strength.

Reinforcement materials		N	Subset for alpha = 0.05		
			1	2	3
Tukey HSDa	Fiberglass	3	0.0500		
	Control	3	0.0567		
	Carbon-Kevlar Fiber	3	0.1613	0.1613	
	Carbon Fiber	3		0.3800	0.3800
	Cement	3			0.4767
	Sig		0.652	0.121	0.752

Overall, yield strength was significantly different among the five socket reinforcements, F (4,10) = 11.572, p < 0.05. Post hoc testing revealed significant differences between different categories of sockets with fiberglass (M = 0.05, SD = 0.02), control (M = 0.06, SD = 0.02), and carbon-Kevlar fiber (M = 0.16, SD = 0.14) exhibiting the lowest yield strength, followed by carbon-Kevlar fiber (M = 0.16, SD = 0.14) and carbon fiber (M = 0.38, SD = 0.16) having intermediate yield strength. The highest yield strength category is the carbon fiber (M = 0.38, SD = 0.16) and cement (M = 0.47, SD = 0.06) pair. These findings indicate that the reinforcement materials significantly impact the yield strength of the prosthetic leg sockets, with carbon and cement exhibiting the highest levels of strength.

#### Young’s modulus (E)

**Figure 16:**
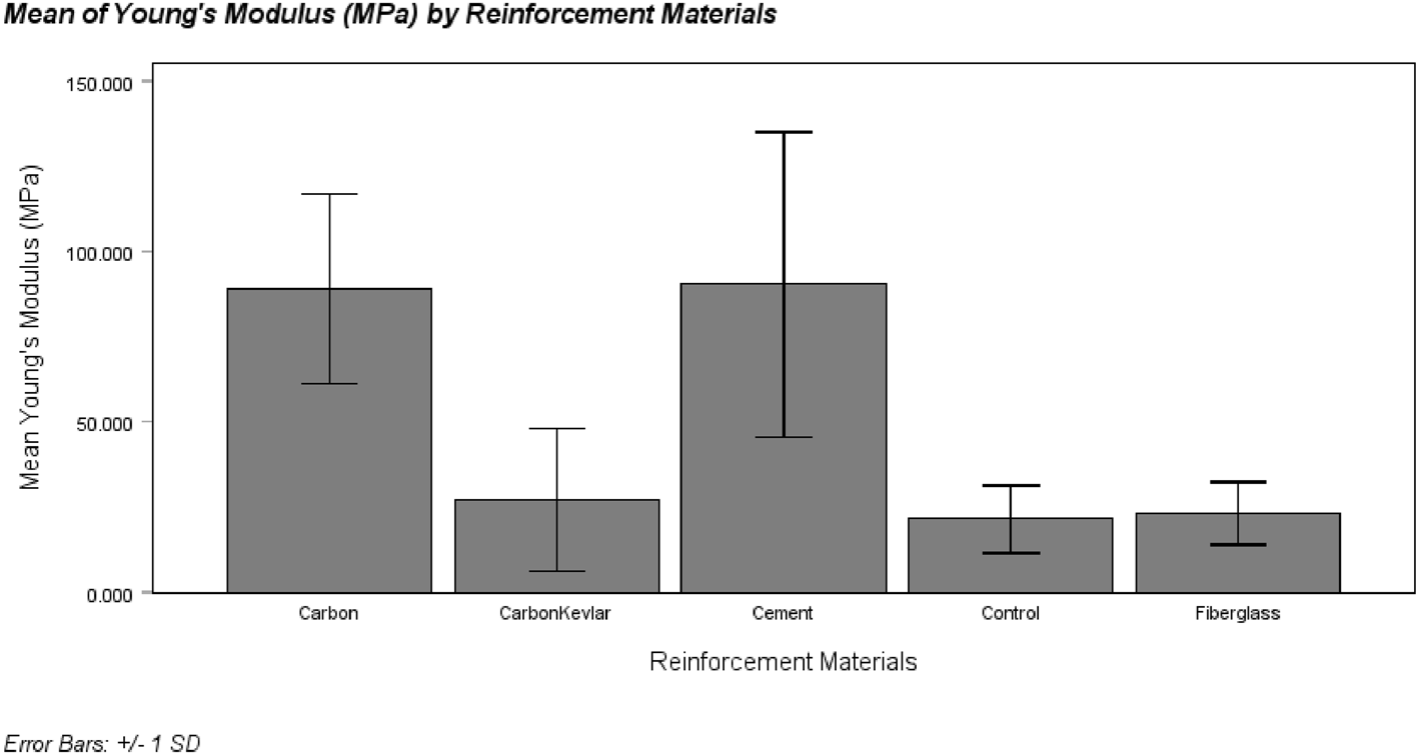
Mean of Young’s modulus for different types of reinforced sockets.

Figure 16 displays the mean values of Young’s modulus for different reinforcement materials used in the sockets. In descending order, the reinforcement materials’ Young’s modulus are cement, carbon, carbon-Kevlar, fiberglass, and control. The relatively large error bars associated with the data points are worth noting, indicating potential issues related to sample size, quality, and testing errors. The standard deviation chosen for the error bars is 1. The ANOVA Table 7 below indicates significant differences in Young’s Modulus among the group means (p < 0.05). Similar to yield strength, Table 8 presents Tukey’s Honest Significant Difference (HSD) test results, categorizing the multiple groups within Young’s Modulus for the reinforcement materials.

**Table 7:** One-way ANOVA for Young’s modulus.

	Sum of squares	df	Mean square	F	Sig
Between groups	15,611.278	4	3902.819	5.734	0.012
Within groups	6806.189	10	680.619		
Total	22,417.467	14

**Table 8:** Tukey’s honest significant difference (HSD) for Young’s modulus.

Reinforcement materials		N	Subset for alpha = 0.05
			1
Tukey HSDa	Control	21.5333	21.5333
	Fiberglass	23.2350	23.2350
	Carbon-Kevlar fiber	27.1733	27.1733
	Carbon fiber	89.1100	89.1100
	Cement	90.3333	90.3333
	Sig	0.055	0.055

While there was a significant difference in Young’s Modulus among the five socket reinforcements, as indicated by F (4,10) = 5.734, p < 0.05, post hoc testing did not reveal any significant variations or distinctions between the groups based on the single alpha subset. This suggests that the observed differences in socket means might be due to random chance rather than actual group differences^41^. Therefore, although the means of the carbon fiber and cement sockets are relatively higher than the other three, these differences lack statistical significance. It is important to note that yield strength and Young’s modulus are related, which could explain the similar mean results between these two groups.

### Scanning electron microscopy (SEM)

SEM analysis was performed in a single session for the fiber reinforcement and surface coating groups. It is worth mentioning that various magnification levels were utilized because fibers were more visible at lower magnifications than at those exceeding 1 k.

#### Carbon fiber

**Figure 17:**
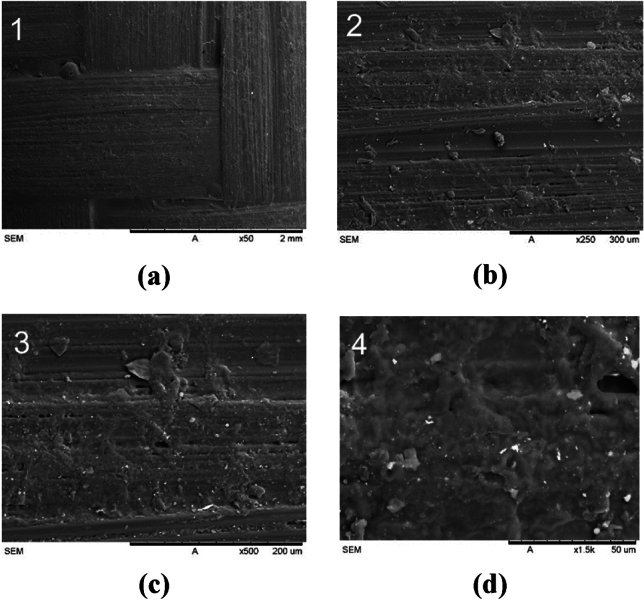
Scanning electron microscopy (SEM) images of the carbon fiber reinforced socket at various magnifications: (**a**) × 50; (**b**) × 250; (**c**) × 500; (**d**) × 1.5 k.

The SEM images in Fig. 17 revealed distinct features for each material, providing insights into their structure and interaction with the surrounding matrix. SEM analysis of carbon fiber showed a uniform surface, indicating a consistent fiber morphology. This uniformity contributes to carbon fiber-filled cement materials’ mechanical properties and electrical conductivity^42^.

#### Carbon-Kevlar fiber

**Figure 18:**
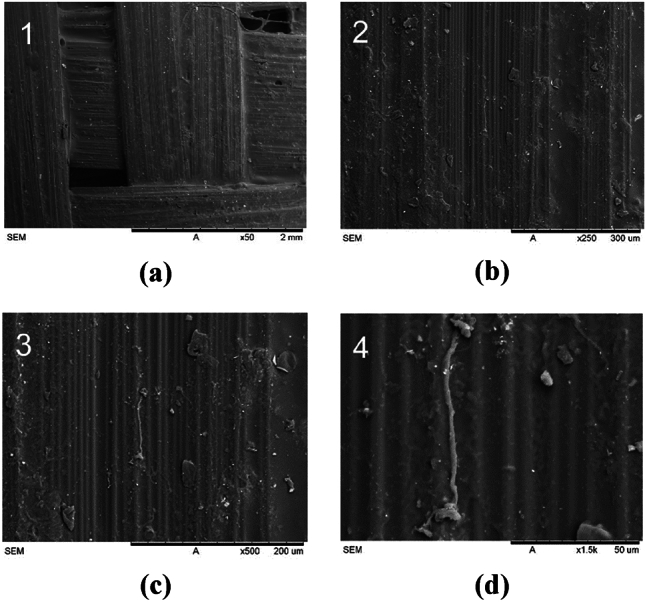
Scanning electron microscopy (SEM) images of the carbon-Kevlar fiber reinforced socket at various magnifications: (**a**) × 50; (**b**) × 250; (**c**) × 500; (**d**) × 1.5 k.

On the contrary, the carbon-Kevlar fiber in Fig. 18 showed a somewhat sparse distribution, indicating fluctuations in fiber density and dispersion^43^. These sparse characteristics of carbon-Kevlar fibers can affect the mechanical properties and overall performance of composite materials.

#### Fiberglass

**Figure 19:**
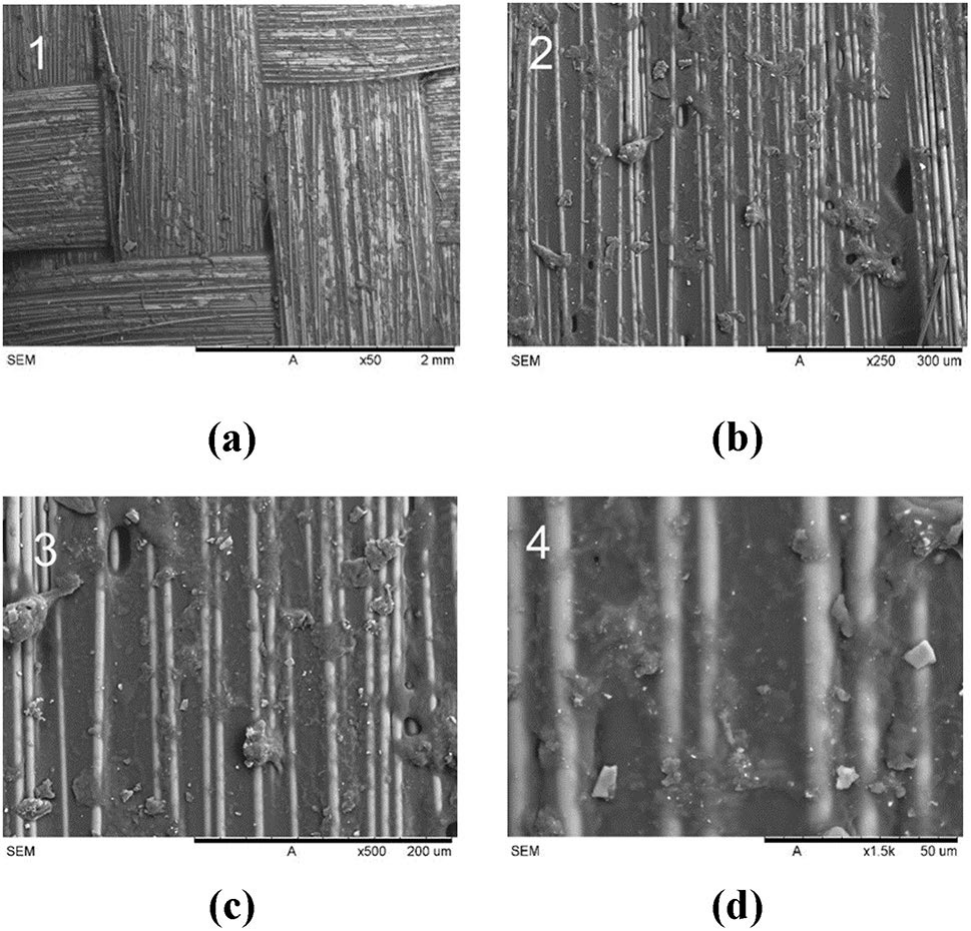
Scanning electron microscopy (SEM) images of the fiberglass reinforced socket at various magnifications: (**a**) × 50; (**b**) × 250; (**c**) × 500; (**d**) × 1.5 k.

Figure 19 shows fiberglass reinforcement with a somewhat rough appearance, suggesting the existence of surface irregularities and roughness. This roughness improvesthe bond between the fibers and the matrix, resulting in increased compressive strength in composite materials^44^

#### Cement

**Figure 20:**
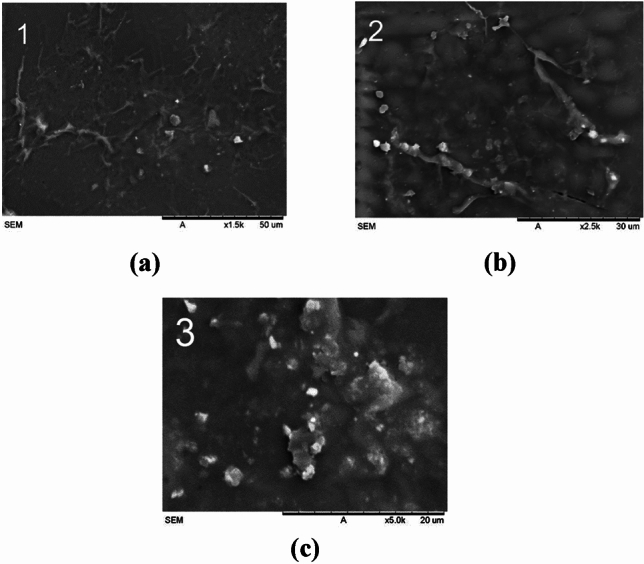
Scanning electron microscopy (SEM) images of the cement-reinforced socket at various magnifications: (**a**) × 1.5 k; (**b**) × 2.5 k; (**c**) 5 k.

SEM analysis of the cement, as seen in Fig. 20, displays a uniform surface, likely due to its application as a sprayed material. This consistent surface uniformity enhances cement’s performance as a reinforcement material ^42^.

#### Control

**Figure 21:**
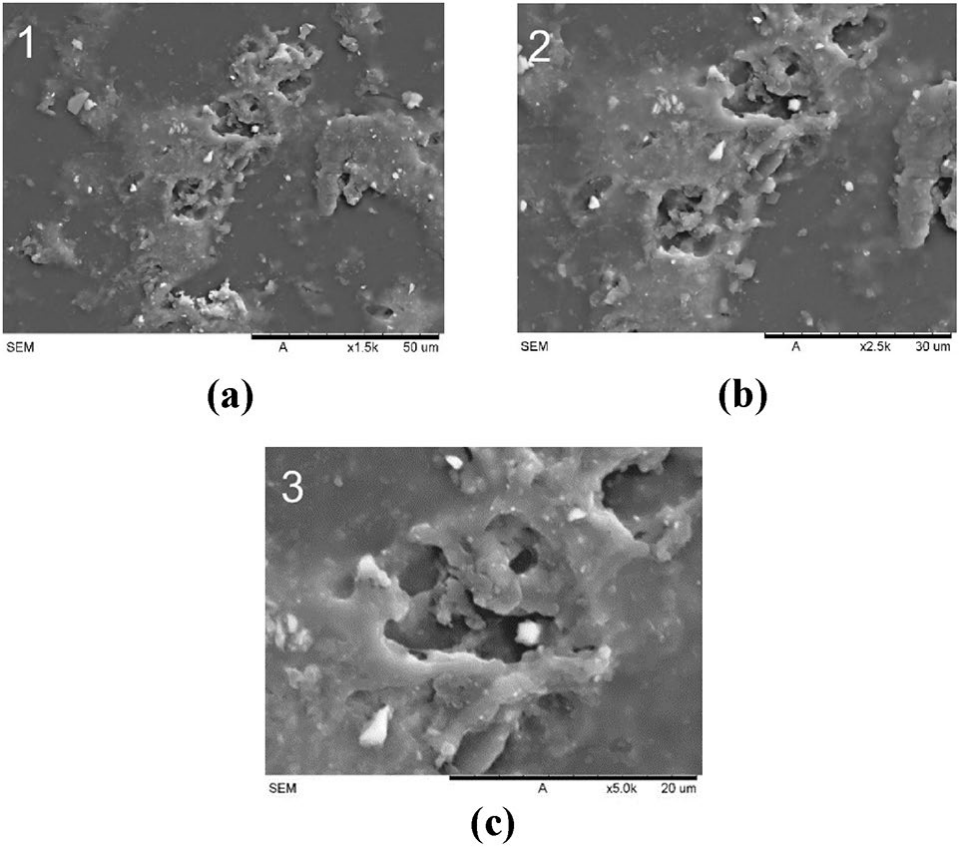
Scanning electron microscopy (SEM) images of the control socket at various magnifications: (**a**) × 1.5 k; (**b**) × 2.5 k; (**c**) 5 k.

SEM images of the control socket in Fig. 21 exhibit a patchy appearance, suggesting surface variations within the control samples. This patchy quality could be due to various factors, including inconsistent material distribution, leading to non-uniform surface characteristics.

#### Comparative analysis of microstructural properties in reinforced sockets

Table 9 provides an overview of the analysis, which aims to assess and compare the microstructural features and surface morphology of various socket reinforcement materials at a magnification of 1.5 k.

**Table 9:** Microstructural analysis of different types of reinforcement materials.

Reinforcement Material (× 1.5 k)	Uniformity	Roughness	Porosity
Carbon fiber	Excellent uniformity and seamless integration between fiber and matrix	It is smooth and has low roughness compared to other materials except for cement	Low porosity due to the tightly packed fiber and matrix bonding
Carbon-Kevlar fiber	A lower level of uniformity compared to carbon	Less smooth compared to carbon	Higher porosity than carbon and fiberglass
Fiberglass	A lower level of uniformity compared to carbon-Kevlar	It has a very rough appearance relative to other reinforcement materials	Low porosity
Cement	Uniform surface, which can be attributed to its application as a sprayed material	Smoother surface because of spraying application	No visible pores
Control	Inconsistent material distribution, resulting in non-uniform surface attributes	Rougher texture due to manual brush strokes	Uneven brush strokes cause pores to form on the surface

## Discussion

### Stress–strain curve

Evaluating stress–strain curves for various leg socket reinforcement materials reveals intriguing insights into their mechanical properties. Firstly, when considering carbon fiber reinforcement, it is worth noting that reported yield strength and Young’s modulus values from previous studies might not accurately represent the true potential of carbon fiber. The actual properties of carbon fiber could be significantly higher than reported, potentially influenced by experimental conditions and sample-specific factors. Nevertheless, previous study into carbon-fiber reinforced sockets using a PLA and carbon hybrid filament offers valuable insights^6^. Their study demonstrated substantial improvements in longitudinal strength and toughness, with estimated tensile strength ranging from 72–79 MPa. Although their focus was on the pre-processing stage and employed a different reinforcement approach, it underscores the potential benefits of carbon fiber reinforcement for enhancing socket strength and overall durability.

In contrast, another study explored prosthetic arm sockets, finding that carbon-Kevlar and carbon fibers, texalium, and polinet exhibited high strength, reaching 207.18 × 106 N/m^2^^45^. When comparing these findings to those of carbon-Kevlar-reinforced leg sockets, a noticeable difference in yield strength is evident. This discrepancy prompts further investigation to understand the underlying reasons. Similarly, the use of glass-reinforced filaments in socket fabrication was previously investigated^9^. Like carbon fiber reinforcement, their study showed increased strength and toughness along the longitudinal direction, with a reported tensile strength of 59.5 MPa for their glass-reinforced sockets. Compared to our findings with fiberglass-reinforced sockets, these results appear relatively lower. However, it is essential to account for various experimental conditions, including sample preparation and testing protocols, as these variables can significantly impact reported mechanical properties.

Regarding cement as a reinforcement material for sockets, direct comparisons are challenging due to limited research in this area. However, a study focused on a concrete application revealed that combining PLA and cement can enhance its mechanical properties^46^. While these studies address distinct fields, they aim to identify novel material combinations to improve strength and performance, offering valuable insights for future research and optimization. Lastly, a previous study on conventional and 3D-printed PLA transtibial leg sockets raises intriguing questions regarding the control socket group^12^. Their sockets exhibited higher ultimate strength, surpassing the values observed in our post-processed PLA + sockets. This discrepancy is noteworthy because PLA + is generally expected to be more durable than PLA. It suggests that our post-processing techniques, such as reinforcement and surface coatings, may not have yielded the exact extent of improvement in ultimate strength as observed in the conventional and 3D-printed PLA sockets. Understanding the specific factors contributing to this difference, such as fabrication techniques and testing conditions, warrants further investigation.

In summary, evaluating stress–strain curves across various leg socket reinforcement materials highlights the potential for significant improvements in strength and durability. However, these findings also underscore the complexity of material interactions and the importance of considering experimental conditions when interpreting results and comparing studies. Further research is crucial to unlock the full potential of these reinforcement materials in enhancing prosthetic leg socket performance.

### Yield strength (σY)

A comprehensive comparative analysis was undertaken to address the research questions posed in the preface, employing a combination of visual representations using bar charts, statistical tests such as One-Way ANOVA, and post hoc testing. These analytical methods were the foundation for assessing and contrasting the strength characteristics of the diverse reinforcement materials used in the prosthetic leg sockets.

In summary, the comparative analysis highlighted significant differences in yield strength among the five socket reinforcements, as verified by statistical analysis F (4,10) = 11.572, p < 0.05. The subsequent post hoc testing unveiled clear distinctions among the different categories of sockets. Fiberglass, control, and carbon-Kevlar fiber emerged with the lowest yield strength values, while carbon-Kevlar fiber and carbon fiber exhibited intermediate strength levels. Notably, the carbon fiber and cement pair demonstrated the highest yield strength.

The variations in yield strength results among the reinforcement materials can be directly attributed to their distinctive material properties. Carbon fiber and carbon-Kevlar, renowned for their remarkable strength and stiffness, significantly enhance the sockets' yield strength^22^. In contrast, fiberglass, with its well-balanced blend of strength and flexibility, lends specific yield strength characteristics to the sockets^47^. Cement, while less commonly explored as a reinforcement material for sockets, appears to hold promise, as its inherent properties could provide an added strength layer to the composite structure^46^.

This analysis emphasized the critical role played by the choice of reinforcement material in determining the yield strength of prosthetic leg sockets. Carbon and cement, in particular, emerge as compelling options due to their capacity to elevate socket strength. However, these findings also highlight the importance of considering material properties when selecting reinforcement materials, as each material brings unique characteristics to the composite structure. In terms of yield strength, cement exhibited a significant advantage over the other materials, with approximately 20.28% higher yield strength compared to carbon, 66.05% higher compared to carbon-Kevlar, 89.57% higher compared to fiberglass, and 88.05% higher compared to the control group.

### Young’s modulus (E)

Although there was a notable difference in Young’s Modulus among the five socket reinforcements, as denoted by statistical analysis (F (4,10) = 5.734, p < 0.05), post hoc testing failed to reveal any significant distinctions or variations between the groups when employing a single alpha subset. This outcome hints that the observed differences in the means of the sockets might be more attributed to random chance rather than actual group distinctions^41^. Therefore, these disparities lack statistical significance despite carbon fiber and cement sockets showing relatively higher means than the other three groups. While it's acknowledged that Young's modulus and yield strength were derived from the same stress–strain curve, indicating some inherent connection, it is also important to consider that this correlation might be more coincidental than significantly meaningful.

Moreover, it is essential to recognize that the results stemming from axial compression tests might not necessarily reflect the typical or average properties of the reinforced materials under ISO 10,328 loading conditions^48,49^. Therefore, further studies should be conducted to gain a more comprehensive understanding of the mechanical behavior and performance of these sockets. These studies should encompass larger sample sizes and diverse experimental conditions, providing a more encompassing perspective on the mechanical attributes of these reinforced materials. In terms of Young’s modulus, cement showcased superior performance, with approximately 1.35% higher modulus compared to carbon, 69.86% higher compared to carbon-Kevlar, 74.20% higher compared to control, and 76.15% higher compared to the fiberglass group. The higher values in cement-reinforced sockets indicate that they have better load-bearing capabilities and stiffness than other materials, highlighting the importance of reinforcement material selection in determining socket mechanical performance.

### Scanning electron microscopy (SEM)

The SEM findings offer valuable insights into the microstructure and surface at-tributes of the reinforcement materials integrated into the sockets. When considered alongside the yield strength and Young's modulus data, these results suggest that the consistency and distribution of the reinforcing materials, as well as their adhesion to the matrix, play pivotal roles in shaping the mechanical properties and overall strength of the sockets^50^. Among these materials, cement emerges as the most effective in terms of reinforcement. Its uniform surface and even material distribution set it apart, followed by carbon fiber, carbon-Kevlar fiber, fiberglass, and finally, the control group. The SEM analysis further provided insights into the microstructural properties of the reinforcement materials, including uniformity, roughness, and porosity.

### Limitation of study

In this study, several limitations of study have been considered due to the several conditions where this could not be avoided in any research and must based on the current scope as demonstrated by many previous researchers^5,51,52^. First and foremost, this study did not investigate the cost effectiveness in using 3D printing protocol for the prosthetics leg socket development. However, future study on the relationship between cost effectiveness and strength of the 3D-printed prosthetics leg socket could be done in the future for better understanding. Nevertheless, the current study is only focus on the mechanical properties and microstructural of the 3D-printed socket reinforcement with different materials of cement, carbon fiber, carbon-kevlar fiber and fiberglass. The second limitation of the study is prosthetic design where there was only one design involved. Since the main aims of the study is to investigate the different materials of reinforcement, therefore, other parameters such as prosthetics design, printing parameter, geometry of patients, and loading condition were set constant. As far as authors concerned, the design is also important for making better prosthetics leg as demonstrated by previous studies^5,51,53^. Nevertheless, the reinforcement materials are also important to be considered to make sure the leg could be last-longer than usual 3D-printed part. Based on the results from this study, cement shows a promising outcome to increase the mechanical properties of the 3D-printed sockets when tested according to ISO 10,328.

## Conclusions

The comparative analysis of the different reinforcement materials used in the prosthetic leg sockets revealed significant variations in yield strength and Young’s modulus. The cement-reinforced sockets demonstrated the highest values for both yield strength and Young’s modulus. These findings contribute to the understanding of the post-processing process for 3D-printed prosthetic leg sockets and provide a basis for future optimizations and advancements in this field. Further research can focus on refining the fabrication techniques, exploring alternative reinforcement materials, and investigating the long-term durability and functionality of the reinforced sockets. Overall, this study opens up new possibilities for improving the design and performance of prosthetic leg sockets, ultimately enhancing the quality of life for amputees.

## Data Availability

The necessary data used in the manuscript are already present in the manuscript.
